# Supplementary material for: Transcriptome Analysis Reveals an Essential Role of Exogenous Brassinolide on the Alkaloid Biosynthesis Pathway in Pinellia Ternata
Source: Int J Mol Sci. 2022 Sep 17;23(18):10898. doi: 10.3390/ijms231810898 (PMC9501358; doi:10.3390/ijms231810898)
Supplement: Supplementary file 1 [file ijms-23-10898-s001.zip › ijms-1895602-supplementary.pdf]

Supplementary Figures and Tables:

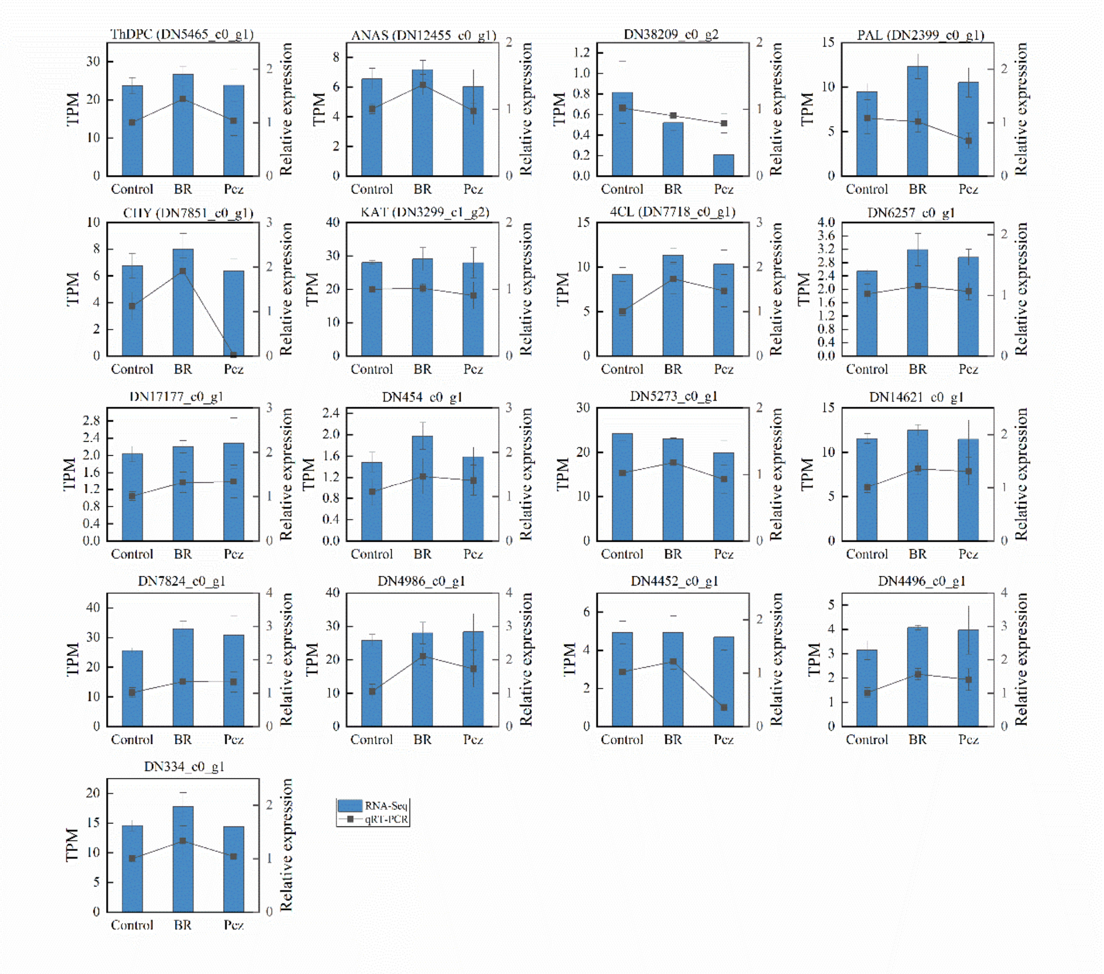

Supplementary Figure S1 Comparison of the expression patterns of unigens involved in the ephedrine biosynthesis under control, BR, and Pcz treatments by qRT-PCR and RNA-seq. Values are means of three replicates ± SE.

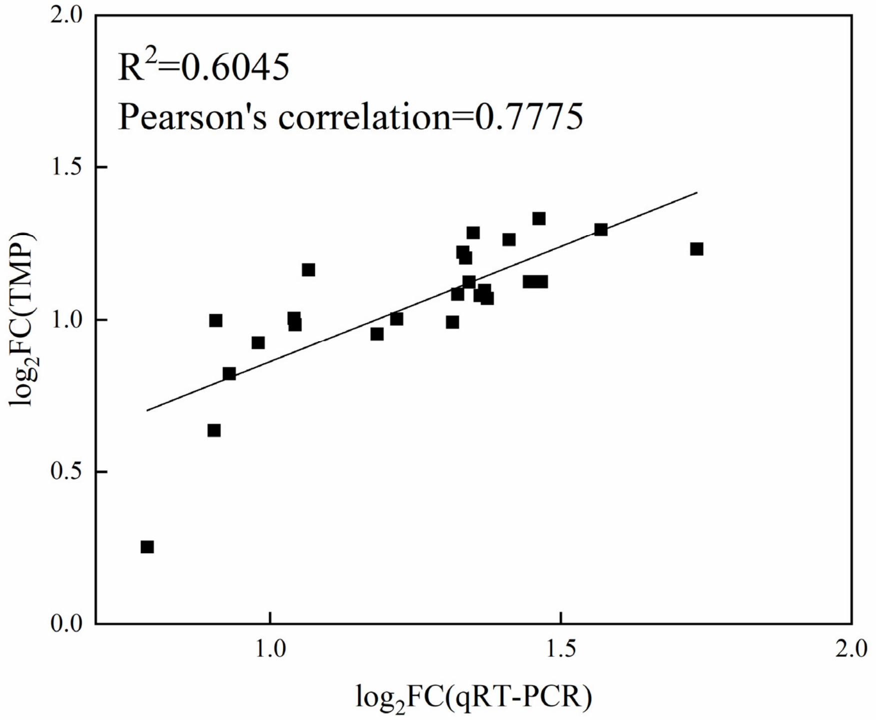

Supplementary Figure S2 Correlation plot between RNA-seq and qRT-PCR for the relative expression of unigenes.

Supplementary Table S1 Summary of annotation of *P. ternata* bulbil unigenes

Database	Number of annotated unigenes	Percentage of annotated
GO	33092	28.66%
KEGG	15065	13.05%
COG	32699	28.32%
NR	38971	33.76%
Swiss-Prot	25696	22.26%
Pfam	27050	23.43%
Total annotated	40992	35.51%
Total Unigenes	115445	100.00%

Supplementary Table S2 Primers used for gene expression analysis by real-time quantitative PCR

Unigene name	Sequence (5’-3’)
DM2399_c0_g1	F: GCCATTTTGTGATCCGTTCTG<br>R: TCCATCTGATCTCCCTCGTC
DN7718_c0_g1	F: TGCAGTGATGGATTTCGTGG<br>R: TTGGAGGTGGCGAGTTTG
DN7851_c0_g1	F: TTCTCAAGGGATTCGGCAAG<br>R: CACAAGAACCCACAAACCAAG
DN3299_c1_g2	F: TGACATTGGTATTGGAGCTGG<br>R: TTCTGAAGTGATGCCCATAGG
DN38209_c0_g2	F: TGATTACTGAACGTTGCAGGG<br>R: ACCTCTTCACCGTATTTGTCG
DN12455_c0_g1	F: GTTTGGGTGCTATGGGTTTTG<br>R: GATTCTCCACCCGTATCATAGC
DN5465_c0_g1	F: GTTCAACTGCCAGAAACTTCG<br>R: ATCACTCGCTTGTCCTTGG
DN7824_c0_g1	F: TCTGGCTCTGCTATGTGTTG<br>R: TTTACGTTGTCACCCCTGTAG
DN14621_c0_g1	F: CCCCTGAACATACCCTTTATCTG<br>R: GTGTCACCCGTCATATCATGTAG
DN4452_c0_g1	F: TGCAAACTCCATAGCAGGG<br>R: AATCTTCACTATGTCAGCCCC
DN17177_c0_g1	F: GTTGCTCAAAGCTTCGGAAAG<br>R: GTATTTGCTCCCTGGACTGG
DN6257_c0_g1	F: GTTGCAGGGTGAAGTTTGTC<br>R: TTGCCTTCCGTTGATCTCC
DN334_c0_g1	F: CACAACCCTCCACAGTGATAG<br>R: GCCAGATGTACAAGTCTCCAG
DN4986_c0_g1	F: GAACAGCTCTAGGAACTACGC<br>R: ACAAAACACCGTAACCAGGG
DN4496_c0_g1	F: AGGAGAGGGTAGAAATGAAAGC<br>R: TCCCCATAAAGATCAGCAACC
DN5273_c0_g1	F: GAGATGTGGGAGAGGACTTTG<br>R: TGTTTAGGAGCGGCAAGATAC
DN454_c0_g1	F: GCGGCATCTTAAGGGATCTG<br>R: CCTCTTCCTGCTCCTGTTG
Pt18S	F: CGCATATAAATAAACGGAGGAA<br>R: GACGCTTCTACAGACTACA
